# Various combinations of living and deceased donors for lung retransplantation—a single institutional retrospective study

**DOI:** 10.1093/icvts/ivae010

**Published:** 2024-01-16

**Authors:** Akihiro Ohsumi, Satona Tanaka, Yoshito Yamada, Yojiro Yutaka, Masatsugu Hamaji, Daisuke Nakajima, Hiroshi Date

**Affiliations:** Department of Thoracic Surgery, Kyoto University Hospital, Kyoto, Japan; Department of Thoracic Surgery, Kyoto University Hospital, Kyoto, Japan; Department of Thoracic Surgery, Kyoto University Hospital, Kyoto, Japan; Department of Thoracic Surgery, Kyoto University Hospital, Kyoto, Japan; Department of Thoracic Surgery, Kyoto University Hospital, Kyoto, Japan; Department of Thoracic Surgery, Kyoto University Hospital, Kyoto, Japan; Department of Thoracic Surgery, Kyoto University Hospital, Kyoto, Japan

**Keywords:** Lung transplantation, Lung retransplantation, Chronic lung allograft dysfunction, Living donor lung transplantation

## Abstract

**OBJECTIVES:**

Lung retransplantation has been performed as a treatment option mainly for chronic lung allograft dysfunction; however, the outcomes of lung retransplantation have been reported to be worse than those of primary lung transplantation. Because of the scarcity of deceased donors in our country, our lung transplant experience includes both living and deceased donors. Therefore, we have experienced lung retransplantation cases with various combinations of living and deceased donors. The aim of this study was to explore technical pitfalls and outcomes of lung retransplantation in this unique environment.

**METHODS:**

We performed 311 lung transplantation procedures between April 2002 and October 2022. Eight lung retransplantation cases (2.6%) were analysed retrospectively.

**RESULTS:**

At lung retransplantation, the age of the recipient patients ranged from 11 to 61 years (median, 33 years). The combinations of donor sources (primary lung transplantation/lung retransplantation) were as follows: 2 living/living, 2 deceased/living, 3 living/deceased and 1 deceased/deceased. Seven of 8 patients received lung retransplantation for chronic lung allograft dysfunction. Hospital death occurred in 2 patients (25.0%). The 1-, 3- and 5-year survival rates after lung retransplantation (*n* = 8) were 75.0%, 75.0% and 75.0%, respectively, while those after primary lung transplantation (*n* = 303) were 92.8%, 83.4% and 76.4%, respectively (*P* = 0.162).

**CONCLUSIONS:**

Lung retransplantation with various combinations of living and deceased donors is a technically difficult but feasible procedure with acceptable outcomes.

**Figure:**
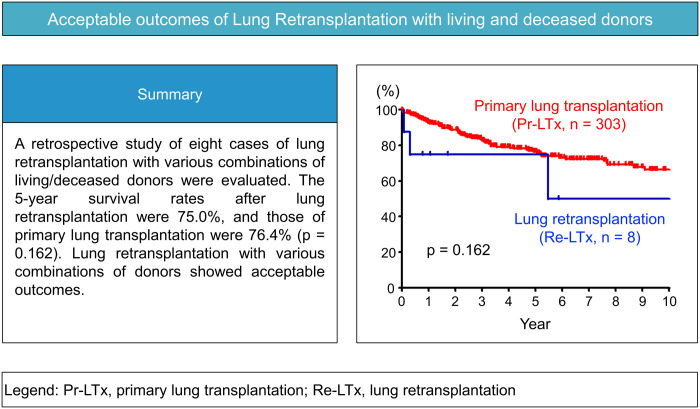

## INTRODUCTION

According to the registry report of the International Society for Heart and Lung Transplantation, the post-transplant survival curve has not improved since chronic lung allograft dysfunction (CLAD) remains a hindrance to survival [1]. Lung retransplantation (Re-LTx) is performed as a treatment option for CLAD; however, the outcomes of Re-LTx have been reported to be worse than those of primary lung transplantation (Pr-LTx) and have not improved in this decade [1].

Historically, lung transplantation (LTx) occurred mainly from living donors rather than deceased donors due to the severe shortage of deceased donors in our country. Living-donor lobar LTx (LDLLTx) is already an established procedure [2]; the advantage of LDLLTx is the ability to prepare and perform the LTx as soon as possible. On the other hand, the disadvantages are that donations should be from healthy donors, and the grafts are relatively small for adult recipients. In addition, various techniques and strategies for LDLLTx have been reported, especially for size mismatch issues such as single lobe transplantation [3], native upper lobe-sparing transplantation [4] and right-to-left inverted transplantation [5]. These techniques in primary or repeat transplantations can significantly affect procedures and outcomes. We have experienced Re-LTx cases, including LDLLTx for primary LTx or Re-LTx, which have different techniques and pitfalls from deceased donor lung transplantation (DDLTx); therefore, we investigated the outcomes in our institution.

## PATIENTS AND METHODS

In our institution, 311 LTxs occurred from April 2002 to October 2022: 191 cases with deceased donors, 119 cases with living donors and 1 case from both a deceased and a living donor. Among these populations, all Re-LTx cases were retrospectively analysed. All data were analysed as of January 2023 according to the patient charts. The survival rates were compared after Re-LTx and Pr-LTx of the other 303 cases.

### Statistical analysis

Continuous data are presented as medians with interquartile ranges. Kaplan–Meier curves and the log-rank test were used to demonstrate and compare overall survival time. Data analysis was performed using GraphPad Prism 5.01, and statistical significance was set at *P* < 0.05.

### Ethical statement

The ethics committee of Kyoto University Hospital approved the study protocol (approval number: R2389-3; approval date revised: 19 May 2022). All patients provided written informed consent for inclusion in this study.

## RESULTS

Among all LTx cases, 8 Re-LTx procedures (2.6%) were performed at our institution and are summarized in Table 1. At the primary LTx, the median age of the recipient patients was 28.5 (18.8–36.3) years, and at the Re-LTx, their median age was 33.0 (24.8–39.3) years. The combinations of donor sources of Pr-LTx/Re-LTx were living/living in 2 cases, 3 living/deceased, 2 deceased/living and 1 deceased/deceased. One case involved a paediatric patient who underwent unilateral LTx from a living donor and contralateral LTx from another living donor for progressive severe primary graft dysfunction (PGD) 17 days after Pr-LTx. For the remaining 7 cases, Re-LTx was performed to treat CLAD after Pr-LTx, and the interval between Pr-LTx and Re-LTx was 760–3724 days.

**Table 1:** Operative procedures and outcomes of lung retransplantation

Case	Donor sources (Pr-LTx/Re-LTx)	Age	Sex	Cause of Re-LTx	Operative side (Pr-LTx/Re-LTx)	Special procedure	Pr-LTx to Re-LTx Interval (days)	Outcome After Re-LTx
1	Living/living	37	F	CLAD (BOS)	Bilateral/bilateral		1029	10 years 7 months alive
2	Living/living	12	F	PGD	Right/left	Removal of left graft	17	3 months dead
3	Deceased/living	31	F	CLAD (BOS)	Bilateral/bilateral	Sparing both upper lobes	1929	1 month dead
4	Deceased/living	35	F	CLAD (BOS)	Right/bilateral		1447	5 years 6 months dead
5	Living/deceased	29	M	CLAD (BOS)	Bilateral/left		2900	1 year alive
6	Living/deceased	46	F	CLAD (BOS)	Left/right		1082	5 years 10 months alive
7	Living/deceased	11	F	CLAD (RAS)	Right/bilateral	Downsizing both lower lobes	760	9 months alive
8	Deceased/deceased	61	M	CLAD (RAS)	Right/left		3724	1 year 8 months alive

BOS: bronchiolitis obliterans syndrome; CLAD: chronic lung allograft dysfunction; F: female; M: male; PGD: primary graft dysfunction; Pr-LTx: primary lung transplantation; RAS: restrictive allograft syndrome; Re-LTx: lung retransplantation.

### Living/living

#### Case 1

The details have been reported previously [6]. A 34-year-old woman with idiopathic pulmonary fibrosis underwent LDLLTx bilaterally. Eleven months later, the patient was diagnosed as having antibody-mediated rejection of the right lung donated by her husband and treated with plasmapheresis and high-dose intravenous immunoglobulin, followed by rituximab. Her respiratory state gradually deteriorated and the left lung, which her mother donated, revealed unilateral bronchiolitis obliterans syndrome (BOS). She was listed for Re-LTx 2 years after LDLLTx; later, she developed bilateral BOS. Her respiratory condition deteriorated, and we performed a re-LDLLTx donated by her brother and sister 10 months after she was on the waiting list. She is currently doing well without oxygen supplementation, 10 years after re-LDLLTx.

#### Case 2

A 12-year-old girl was diagnosed with transposition of the great arteries and underwent pulmonary artery banding and atriotomy at the age of 5. However, pulmonary hypertension (PH) persisted, and the patient received Jatene’s operation and closure of atrial and ventricular septal defects at the age of 6. Despite combination therapy, her PH status worsened, and she received right unilateral LDLLTx. After transplantation, the graft showed severe PGD, and she underwent contralateral LDLLTx from another living donor 17 days after Pr-LTx. Unfortunately, the retransplanted left lung became congestive resulting in graft extraction, and the patient died of multiple organ failure 3 months after Re-LTx.

### Deceased/living

#### Case 3

A 19-year-old woman presented with acute promyelocytic leukaemia, an anterior mediastinal tumour and right hilar and supraclavicular lymph node enlargement. She was treated with chemotherapy followed by irradiation for residual tumour. At the age of 20 years, she received bone marrow transplantation from a bank donor and developed bronchiolitis obliterans 10 months later. She was considered a candidate for DDLTx and underwent bilateral DDLTx at the age of 26 years. Five years after DDLTx, the patient was diagnosed with BOS-type CLAD. Respiratory function progressively deteriorated, and it was thought difficult to wait for Re-LTx from deceased donor; therefore, LDLLTx was planned. At the age of 31 years, she received bilateral Re-LDDLTx, sparing both upper lobes as the first donor lungs had no signs of infection. Unfortunately, the patient developed PGD and died on postoperative day 29.

#### Case 4

A 31-year-old woman underwent right DDLTx for interstitial pneumonia due to systemic sclerosis. Later, the transplanted right lung revealed BOS, and her left native lung was exacerbated with repeated pneumothorax requiring surgical intervention. Four years after the Pr-LTx, the patient underwent bilateral LDLLTx and returned to normal life. Unfortunately, the patient developed CLAD and died of chronic heart failure, likely due to systemic sclerosis and renal failure 5 years and 6 months after the Re-LTx.

### Living/deceased

#### Case 5

**Figure 1:**
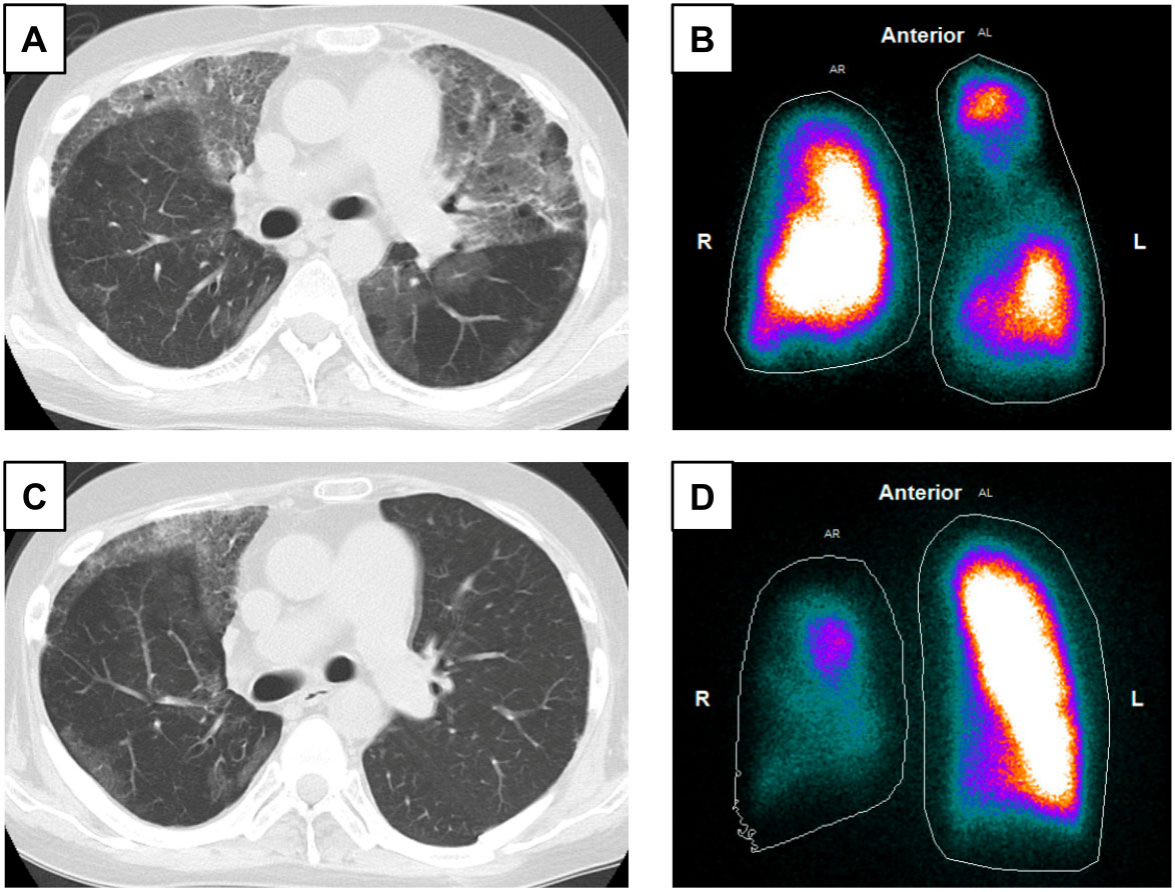
Chest images 9 years after bilateral living donor lobar lung transplantation with sparing bilateral native upper lobes for idiopathic pulmonary arterial hypertension (**A**, **B**). Chest computed tomography (CT) showing organizing pneumonia in the spared native upper lobes and bronchiolitis obliterans in the transplanted lower lobes (**A**). Perfusion scan showing reduction of perfusion in both upper lung fields: 58% of the right and 42% of the left lungs (**B**). Chest images 9 months after left lung retransplantation from a deceased donor (**C**, **D**). Chest CT showing clear left lung field (**C**). Perfusion scan showing deviation of perfusion to the left transplanted lung: 14% of the right and 86% of the left lungs (**D**).

A 21-year-old man with idiopathic pulmonary arterial hypertension (IPAH) underwent LDLLTx-sparing bilateral upper lobes because of an estimated lower size matching. The patient developed organizing pneumonia in the native upper lobes and BOS in the transplanted lungs (Fig. 1A and B), and his respiratory function gradually deteriorated despite steroid therapy. He was considered a candidate for Re-LTx and listed on the waiting list for DDLTx. Eight years after Pr-LTx, he underwent left unilateral LTx from a deceased donor, and the postoperative course went well (Fig. 1C and D). The patient is alive and well.

#### Case 6

A 43-year-old woman with interstitial lung disease induced by rheumatoid arthritis was assessed for registration on the waiting list for DDLTx. However, her respiratory dysfunction progressed, and LDLLTx was planned 2 months later. She underwent unilateral right-to-left LDLLTx due to the lack of a second living donor. One year later, the graft function deteriorated, and she was diagnosed with BOS-type CLAD of the left transplanted lung with exaggeration of the right native lung. She was evaluated and listed for DDLTx. At the age of 46, she underwent right unilateral DDLTx. Although she developed BOS in the transplanted right lung and restrictive allograft syndrome (RAS) in the left transplanted lung, she is alive 5 years after Re-LTx.

#### Case 7

**Figure 2:**
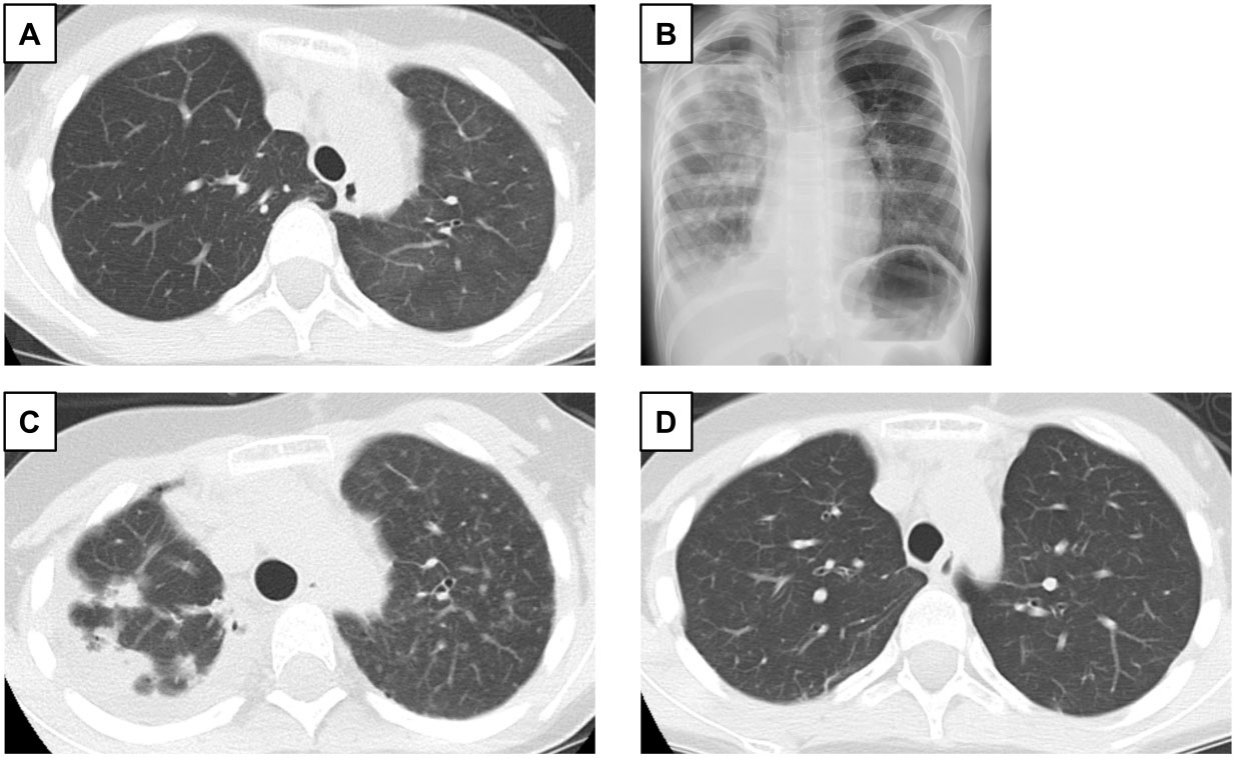
Chest computed tomography (CT) 2 months after right living donor lobar lung transplantation (LDLLTx) for idiopathic pulmonary arterial hypertension showing a right clear transplanted lung and a left native lung filled with slight ground-grass opacities (**A**). Chest X-ray 20 months after right LDLLTx showing broad infiltration in the right transplanted lung with pneumothorax (**B**). Chest CT 20 months after right LDLLTx showing chronic lung allograft dysfunction with subpleural fibrosis of the right lung and exaggerated ground-grass opacities all over the left lung (**C**). Chest CT 6 months after bilateral lung retransplantation from a deceased donor with downsizing bilateral lower lobectomies showing a clear lung field on both sides (**D**).

**Figure 3:**
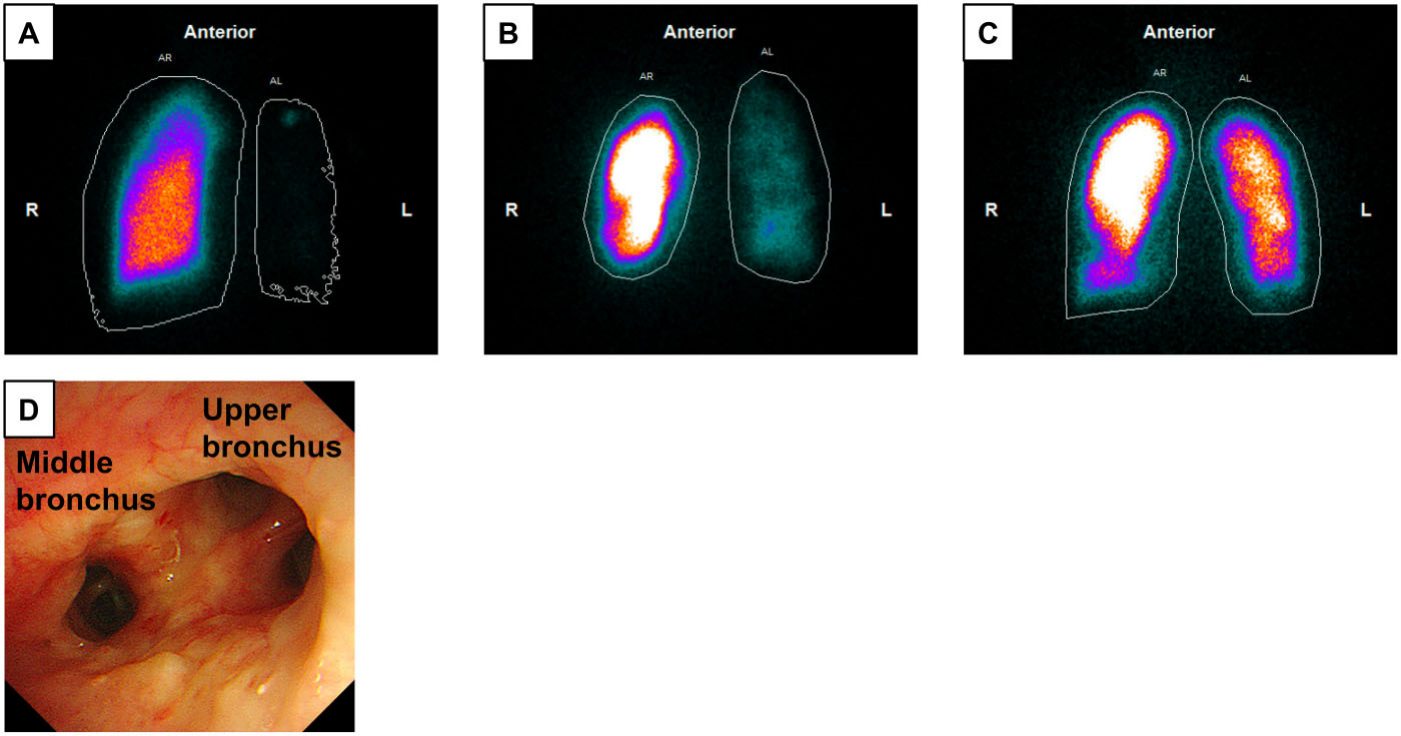
Perfusion scan 2 months after right living donor lobar lung transplantation (LDLLTx) for idiopathic pulmonary arterial hypertension showing deviation of perfusion to the right transplanted lung: 97% of the right and 3% of the left lungs (**A**). Perfusion scan 20 months after right LDLLTx showing deviation to the left native lung: 86% of the right and 14% of the left lungs (**B**). Perfusion scan 6 months after bilateral lung retransplantation (Re-LTx) from a deceased donor showing balanced perfusion: 61% of the right and 39% of the left lungs (**C**). Bronchoscopic findings 4 months after Re-LTx showing good healing of neo-second carina (**D**).

An 8-year-old girl with severe IPAH and an atrial septal defect was referred to our hospital for DDLTx registration. Chest radiography showed cardiomegaly, and cardiac catheterization showed a pulmonary artery pressure of 165/95 mmHg (mean 120 mmHg). Urgent LDLLTx was considered; however, because there was only 1 suitable living donor, right unilateral LDDLTx was performed (Figs 2A and 3A). Seventeen months after LDLLTx, she started to repeat the infection; however, the cause of the infection was not observed via repeated bronchoscopy. The oxygenation and lung imaging of the right transplanted lung gradually worsened (Fig. 2B and C), and the patient was diagnosed with CLAD. The perfusion ratio shifted to the left native lung with IPAH (Fig. 3B), and the left lung image was exaggerated (Fig. 2C). The patient was then evaluated for DDLTx registration and registered on the waiting list. Five days after registration on the organ transplant network, she received bilateral DDLTx for the right lung with CLAD and the left native lung with IPAH. Size matching was +126%, and both lower lobes were entirely resected. The right upper and middle bronchi were anastomosed side-to-side fashion using 4–0 polydioxanone on the back table. This neo-second carina was anastomosed to the recipient’s right main bronchus using 4–0 polydioxanone (Video 1). The donor’s left upper bronchus was anastomosed to the recipient’s left main bronchus. The transplanted lungs worked well (Figs 2D and 3C) and she was discharged 102 days later. Bronchoscopic findings 4 months after LTx showed good healing of the neo-second carina (Fig. 3D).

### Deceased/deceased

#### Case 8

A 51-year-old man with chronic obstructive pulmonary disease who underwent lung volume reduction surgery received right DDLTx. Three years later, the right transplanted lung revealed a RAS type of CLAD, and he was considered a candidate for Re-LTx and was listed on the waiting list. At the age of 61 years, he underwent left unilateral Re-LTx from a deceased donor. Although he needed laparoscopic fundoplication for gastroesophageal reflux disease, the patient is alive and doing well.

#### Survival

**Figure 4:**
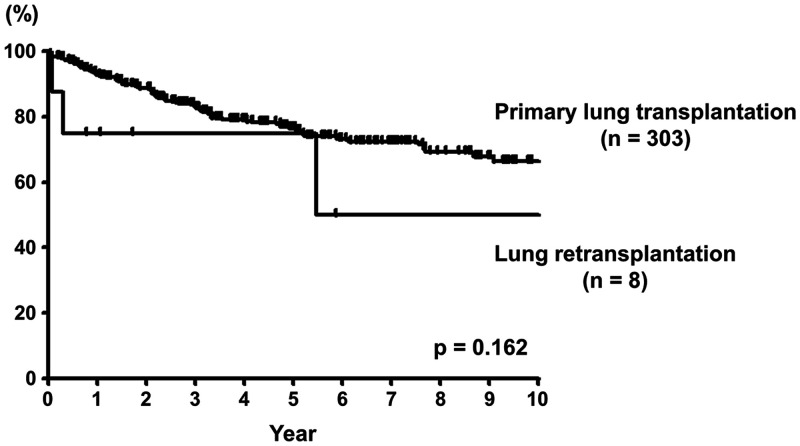
The survival curves after primary lung transplantation and lung retransplantation. The 1-, 3- and 5-year survival rates after lung retransplantation were 75.0%, 75.0% and 75.0%, respectively, and those after primary lung transplantation of the other 303 cases were 92.8%, 83.4% and 76.4%, respectively (*P* = 0.162). Pr-LTx: primary lung transplantation; Re-LTx: lung retransplantation.

The intervals between Pr-LTx and Re-LTx and outcomes after Re-LTx are shown in Table 1. The 1-, 3- and 5-year survival rates after Re-LTx were 75.0%, 75.0% and 75.0%, respectively, and those after Pr-LTx of the other 303 cases were 92.8%, 83.4% and 76.4%, respectively (*P* = 0.162). (Fig. 4). Regarding the cause of death, 2 patients had PGD and died one and 3 months after Re-LTx. One patient had CLAD and died of multiple organ failure 5 years after Re-LTx. The remaining 5 recipients survived, and only 1 progressed to CLAD.

## DISCUSSION

We experienced 8 cases of Re-LTx with different donor sources. In 1998, an International Pulmonary Retransplantation Registry report revealed a 1-year actuarial survival rate of 47% in 230 patients undergoing Re-LTx and identified early graft failure or airway complications and preoperative ventilator dependency as negative predictors of survival [7]. Later, the Hannover group reported 54 retransplants with a 1-year survival rate of 50% for patients with PGD, airway complications, or preoperative ventilator support, in contrast to better outcomes for stable patients with chronic graft failure [8]. A UNOS Registry report based on 604 patients undergoing retransplants from 2004 to 2015 revealed a 5-year survival rate of 35%. An interval of less than 1 year after the first transplant, single lung retransplant, pretransplant ventilator support and multiple retransplants were identified as significant predictors of decreased survival [9]. In our series, only 1 patient received Re-LTx for primary graft dysfunction early after primary LTx, and this patient was lost due to multiple organ failures. Although unilateral LDLLTx can be a lifesaving option for PGD if there is another satisfactory living donor, the outcomes may not be predictable. Besides case 2, we performed Re-LTx in patients with CLAD. In 2011, Sato *et al.* [10] reported a novel form of CLAD after lung transplantation, RAS, which is characterized by restrictive physiology and peripheral lung fibrosis, further demonstrating the poorer prognosis of RAS compared with BOS [11]. Verleden *et al.* [12] evaluated the effect of CLAD phenotypes on survival after Re-LTx for CLAD and demonstrated that survival after Re-LTx for RAS was worse than that for BOS. In this current series, we have 7 retransplant patients with CLAD, consisting of 5 cases of BOS types and 2 cases of RAS types, which might explain the more favourable survival rate than previous reports.

### Role of living-donor lobar lung transplantation in lung retransplantation

To the best of our knowledge, only 1 study has reported on LDLLTx in Re-LTx [13]. Huddleston *et al.* [13] reported Re-LTx in children, wherein 4 instances of living donors were used for the Re-LTx procedure; they reported that the most striking difference between these procedures and deceased donor re-LTx was the shorter donor lung ischaemic time (99.5 and 123.3 min for the 2 lungs from living donors and 251 and 293 min for the first and second lung from deceased donors, respectively; *P* < 0.01). We experienced similar trends in our study; the ischaemic time for lungs for living donor Re-LTx was 95.0 (92.3–119.5) min for the first lung procedure (cases 1–4) and 245.0 (183.5–269.0) min for the second lung procedure (cases 1, 3 and 4), while that for lungs for deceased donor Re-LTx was 467.5 (408.5–483.8) min for the first lung procedure (cases 5–8) and 573 min for the second lung procedure (case 7). Deceased donor lungs are transported via public transport in Japan; therefore, the ischaemic times were much longer than those reported by Huddleston *et al.* [13]. As a result, the shorter ischaemic time from an adjacent operation room has a great advantage. Furthermore, living donors should be healthy under strict medical screenings and the quality of the lungs should be better than that of deceased donors, although the graft sizes of living donors are smaller than those of deceased donors. Considering the waiting time on the list for deceased donors, the lung allocation scoring system according to the candidate’s medical urgency is not applied in our country. Retransplant candidates may not need to wait if appropriate living donors can offer grafts, although 2 healthy donors are required. With these advantages, the option of living donor Re-LTx can achieve favourable outcomes, which is equivalent to deceased donor Re-LTx in our program.

### Surgical side and extracorporeal circulation in lung retransplantation

At the time of Re-LTx, patients are expected to have dense pleural and mediastinal adhesions that require meticulous dissection for minimal bleeding. Kon *et al.* examined the outcomes of Re-LTx for patients with a previous unilateral primary LTx. They found that patients who received contralateral unilateral Re-LTx had similar survival at 30 days, 1 year and 5 years to those who received bilateral Re-LTx, but that survival for both was superior to that of patients who received ipsilateral unilateral LTx [14]. In this regard, contralateral Re-LTx appears to be ideal after unilateral LTx, and unilateral Re-LTx appears to be ideal after bilateral LTx. In our series, although cases 1 and 3 needed bilateral Re-LTx after bilateral LTx, cases 6 and 8 underwent contralateral Re-LTx after unilateral Pr-LTx, and cases 4, 5 and 7 needed re-thoracotomy only on the unilateral side.

Regarding the strategy of extracorporeal circulation during LTx, we consider veno-arterial extracorporeal membrane oxygenation (ECMO) when the patient had hypercapnia or pulmonary hypertension before or after general anaesthesia induction, when haemodynamic instability or pulmonary hypertension occurs after clamping of the right or left pulmonary artery, when the graft is relatively small, or when the chest cavity is small, and it is difficult to maintain a good surgical field [15]. In the current series, all cases were performed on veno-arterial ECMO; however, case 3 required cardiopulmonary bypass to control bleeding caused by injury of the pulmonary artery during dissection. ECMO with a smaller dose of heparinization is preferable for Re-LTx. Abdelnour-Berchtold reported that patients requiring ECMO as a bridge to a retransplant had lower overall and CLAD-free survival rates compared to those who had a retransplant without bridging ECMO [16]. Inci *et al.* [17] performed a retrospective multicentre cohort analysis from 10 centres and found that recipients >35 years of age, those bridged on VA-ECMO, those with PGD as a transplant indication, those with an inter-transplant interval <1 year and those with a Zurich donor score >4 have the worst prognosis when bridged on ECLS to Re-LTx and should probably be excluded from Re-LTx. The most common complications in another cohort awaiting Re-LTx were sepsis or coagulopathy [18]. In this situation, LDLLTx is an alternative to bridging ECMO if there are suitable living donors because there is no need to wait on the list.

### Bronchial anastomotic sites in combination with living-donor lobar lung transplantation

Once the chest was opened, and the primary transplanted lungs were dissected from the chest cavity, the next step was to decide how to secure the pulmonary arteries and where to anastomose the pulmonary arteries and bronchi. Meticulous dissection in Re-LTx is necessary to avoid bleeding from proximal pulmonary arteries. However, this may decrease the blood supply for the bronchus from the surrounding tissue, leading to bronchial comorbidities. One of the ways to deal with this issue is to cover the bronchial anastomosis with donor’s and recipient’s pericardium or surrounding connective tissue. Arterial and bronchial anastomoses can be made in Re-LTx at different sites from the Pr-LTx, especially with the combination of donor sources that comprise LDLLTx. If the patient underwent conventional bilateral DDLTx, sparing the upper lobes in Re-LTx from living donors requires anastomosis at different sites, as in case 3. Similarly, if the upper lobes are spared at the primary LDLLTx, the anastomosis will be easier at the main bronchus in Re-LTx from a deceased donor, as in case 5. However, in both cases, the spared upper lobes should not have an infection.

### Double-barrel bronchoplasty at the back table

Airway complications remain a major cause of morbidity and mortality after cardiothoracic transplantation [19]. In addition, Re-LTx for airway complications has a very poor prognosis, as well as that for acute graft failure [8]. Our team generally tries to avoid leaving donor bronchial stumps by lobar-to-lobar bronchial anastomosis and instead leave recipient bronchial stumps, if necessary [20]. In case 7, downsizing lower lobectomies were required. There was no choice to anastomose to the upper and middle lobe bronchi because she had received right unilateral LDLLTx; therefore, we performed double-barrel bronchoplasty in the donor upper and middle lobe graft at the back table and anastomosed to the recipient’s main bronchus. The technique was similar to our previously reported double-barrel bronchoplasty for the donor tracheal bronchus; the neo-upper lobe bronchus created by suturing the apical tracheal bronchus and the upper lobe bronchus of the donor was anastomosed to the upper lobe bronchus of the recipient in a double-barrel fashion [21].

### Limitations

Our study had several limitations. First, it was retrospective and had a small sample size in a single institution. Only the surviving recipients with CLAD on the waiting list could receive Re-LTx because of severe donor organ shortage in our country, so retransplant patients have a selection bias for better survival.

## CONCLUSION

In conclusion, Re-LTx from deceased donors for patients with CLAD has been mainly performed on the contralateral side of the primary unilateral LTx. Upper lobe sparing at Pr-LTx or Re-LTx creates different anastomotic sites, which may alleviate the technical difficulty and bronchial comorbidity. Re-LTx is a viable treatment option for post-transplant recipients with CLAD.

## Data Availability

The data supporting this article cannot be shared publicly because it is protected patient information.

## Notes

Presented at the 58th Annual Congress of the Japan Society for Transplantation, Nagoya, Japan, 15 October 2022, and Transplantation Science Symposium Asian Regional Meeting, Kyoto, Japan, 25 November 2022.

## ABBREVIATIONS

BOS: Bronchiolitis obliterans syndrome
CLAD: Chronic lung allograft dysfunction
DDLTx: Deceased donor lung transplantation
ECMO: Extracorporeal membrane oxygenation
IPAH: Idiopathic pulmonary arterial hypertension
LDLLTx: Living-donor lobar lung transplantation
LTx: Lung transplantation
PGD: Primary graft dysfunction
PH: Pulmonary hypertension
Pr-LTx: Primary lung transplantation
RAS: Restrictive allograft syndrome
Re-LTx: Lung retransplantation
